# Changes in cesarean section rate before and after the end of the Korean Value Incentive Program

**DOI:** 10.1097/MD.0000000000029952

**Published:** 2022-08-19

**Authors:** YouHyun Park, Jae-hyun Kim, Kwang-soo Lee

**Affiliations:** a Department of Health Administration, Yonsei University Graduate School, Wonju, Republic of Korea; b Department of Healthcare Administration, Dankook University, Cheonan, Republic of Korea.

**Keywords:** cesarean section rate, interrupted time series analysis, P4P, risk adjustment, value incentive program

## Abstract

**Background:**

The Korean government implemented a value incentive program providing incentives to providers based on C-section rates, with the rates being publicized. The program ended in 2014 after the administration decided that the effects of the incentive program were limited. In this report, we analyzed changes in C-section rates with the value incentive program.

**Methods:**

The analysis used claim data from Korea’s National Health Insurance. The study period (2011–2016) was divided into two phases: before and after the program. This study included 95 providers that were tertiary or general hospitals having more than 200 deliveries per year during the study period. The dependent variable was the risk-adjusted C-section rate. Independent variables included time and hospital characteristics such as hospital type, district, and ownership. Interrupted time series analysis was performed to analyze the data.

**Results:**

Our results showed that risk-adjusted C-section rates increased immediately after the end of the incentive program for C-sections. The immediate effect of intervention, a change of 1.73% (*P* < .05), was statistically significant, as was the trend after intervention, at 0.21% (*P* < .0001). The slope showed an increase after the intervention to 0.25% per medical institution, which was contrary to the trend of the preintervention decline (negative slope).

**Conclusion:**

Risk-adjusted C-section rates increased immediately after the discontinuation of a value incentive program. Tertiary hospitals showed greater increases in C-section rates than general hospitals after the intervention.

## 1. Introduction

According to the 2017 Organisation for Economic Co-operation and Development (OECD) Health Statistics, the number of C-sections per 1000 births in Korea was 452, which was the second highest after Turkey (531 cases) and 190 cases higher than the average of OECD member countries (264 cases). The number of C-sections per 1000 births in Korea was higher than that for women in the Asia–Pacific region, Japan, Taiwan, or the United States, who have similar obstetric conditions as Korean women. The World Health Organization (WHO) recommends maintaining C-section rates at 10–15%,^1^ but it is increasing globally (including in Korea). WHO also indicated that the negative consequences of cesarean delivery must be limited, especially if C-section rates exceed 7%.^2^ C-section rates above the appropriate level not only increase the prevalence and risk of death for mothers and newborns but also contribute to the inefficient use of medical resources.^3^

Therefore, the Korean government implemented a Value Incentive Program in January 2011 to assess the quality of medical services and provide incentives to hospitals to protect maternal health and optimize C-section rates, ultimately reducing health insurance finances. According to the results of the quality assessment of medical services, the providers were incentivized by adding 1%–5% of the fee to higher-grade institutions and quality improvement institutions, and by applying 1%–5% of the fee reduction to medical institutions below the standard. However, the Value Incentive Program was terminated in January 2014 as the attainment of a certain level of medical quality and the introduction of the Korean Diagnosis-Related Group (KDRG) had diminished the influence of policy.^4^ Currently, only monitoring services have been implemented since 2016.

By disclosing assessment results, the Korean Value Incentive Program forced hospitals to manage quality assessment indicators. However, the rating information is no longer available on the Health Insurance Review and Assessment Service (HIRA) website, a quasi-governmental organization that reviews National Health Insurance (NHI) program claims in South Korea. Furthermore, the C-section rate may be increasing since the program was eliminated.^5^

Previous studies have explored the effects of publicizing assessment results on C-section rates. Ko et al confirmed the average change of cesarean rate decreased by 10.2% after the public disclosure of information and the fluctuation also decreased.^6,7^ Jang et al found that the repeated public releases decreased the cesarean section rate (by 0.81 %).^8^ According to Hong et al, it was confirmed that most clinical indicators (such as the number of hospitalizations, etc.) which are subject to incentives have risen since the implementation of the Korean Value Incentive Program.^9^ However, no one has explored the relationship between the discontinuation of the Korean Value Incentive Program and the subsequent C-section rates.

The purpose of this study was to analyze how hospitals responded to terminating the Korean Value Incentive Program for cesarean delivery. Specifically, this study calculated the risk-adjusted C-section rates for each hospital and analyzed whether they changed after the Korean Value Incentive Program ended.

## 2. Method

### 2.1. Data sources

Data from 72 months (January 2011 to December 2016) were analyzed, with the period divided into two segments: 36 months before and 36 months after January 2014. The study data included the delivery mode, maternal age, region, and health at the birth of women of childbearing age (16–49 years) in tertiary hospitals and general hospitals with more than 200 deliveries per year. Data were retrieved from the customized database of the NHI program.

Delivery was defined according to the ICD-10 code (O80, O81, O82, O83, O84), HIRA procedure code (R3131, R3133, R3136, R3138, R3141, R3143, R3146, R3148, R4351, R4353, R4356, R4358, R4380, R4514, R4516, R4361, R4362, R4517, R4518, R4519, R4520, R4509, R4510, R5001, R5002, R4507, R4508), and HIRA code for Diagnosis Related Groups (DRGs) (O01600, O01601, O01602, O01603, O01700, O01701, O01702).^10^

### 2.2. Measurement of variables

The dependent variable was monthly C-section rates in hospitals, which was adjusted for risk differences among patients from different hospitals. The risk adjustment model was established based on the selected risk factors ^11,12^. The risk-adjusted C-section rates were calculated by dividing the crude rates of C-section in hospitals by the predicted rates of C-section in those hospitals, multiplied by the crude C-section rates of all patients. The predicted C-section rates in hospitals were calculated by dividing the sum of the predicted probability of C-section for a patient by the total number of deliveries.^12,13^

Time and hospital characteristics were used as independent variables. The “time” variable was coded as 1 to 72 to represent the monthly time intervals from January 2011 to January 2016. The Korean Value Incentive Program ended in January 2014. The policy intervention was dummy coded: intervention type = 0 for the 36 months from January 2011 to December 2013, and intervention = 1 for the 36 months from January 2014 to December 2016. The “time after policy” variable represented time since the policy was discontinued. This variable was coded as 0 for times before the policy intervention and by month in numerical order starting with 1 after the policy intervention. The model includes hospital characteristics known to affect the C-section rates.^14^ The hospital type was coded as “Tertiary” = 1 and “General” = 0; the administrative district as “Metropolitan” = 1 and “Province” = 0; and ownership type as “Private” = 1 and “Public” = 0.

### 2.3. Analysis

A risk-adjusted model was established with 17 risk factors selected based on a previous report.^12,13^ The risk-adjustment model was developed in the following order.^10^ First, a univariate analysis was performed to compare the distribution of 188,094 cases of vaginal delivery and 176,258 cases of cesarean section of 364,352 claims for each of the 17 risk factors. Second, selected risk factors were included in multivariate analysis, and the final model was developed based on their statistical significance and regression coefficients. Using this approach, 13 factors were selected as factors for the risk-adjusted model, excluding 4 factors (multiple pregnancy, fetal stress, cord prolapse, and premature membrane rupture).

Because the analysis used time-series data, an interrupted time series analysis was performed using a generalized estimated equation (GEE) considering the correlation that has been repeatedly measured.^15,16^ Details related to autocorrelation were mentioned in Appendix 3, Supplementary Digital Content, http://links.lww.com/MD/G988. GEE is used to estimate the causal model of panel data and is an analytical method that can address time-series data that are difficult to handle in Generalized Linear Model (GLM).^17^ The GEE analysis method is used in medical and health studies with repeated measurements where a phenomenon is commonly evaluated using more than one outcome variable.^18^

Data in this study included 36 months of panel data for which the correlation of error terms within a group could be determined; that is, the GEE was used to consider the autocorrelation of the error terms within each group.^16^ The regression model for the interrupted time series (ITS) analysis used in this study was as follows.^19^


$$\begin{array}{l} Y_{it}=B0+B1*time_{t}+B2*\mathrm{int}ervention_{t}+B3*timeafterinvention_{t}+X_{it}+e_{it}. \end{array}$$

Where *Y* is the monthly C-section rate by a hospital, time is the monthly time flow from 2011 to 2016, intervention is the endpoint of the policy (January 2014), time after the intervention is the monthly time flow after the end of the policy, *X* = is the hospital characteristics, and *e* is the error term. The regression coefficient for the “intervention” variable represents the extent of change in the dependent variable at the time of the policy intervention; thus, the immediate effect of the policy can be seen. The regression coefficient for the “time after policy” variable allows us to assess the long-term effects of the policy through trends in the dependent variables after policy intervention.

This study used SAS 9.4 to identify the risk-adjusted C-section rates and to perform ITS analysis.

## 3. Results

### 3.1. Risk-adjusted C-section rates

All 13 risk factors in the model showed a significant association with C-section delivery, with odds ratios >1.0. The odds ratio was highest for breech malpresentation (56.03), followed by placenta previa (55.15). Diabetes had the lowest odds ratio, 1.10. When all 13 risk factors were used in the risk-adjustment model, the C-statistic was 0.91 and the Hosmer–Lemeshow Chi-square statistic (4472.80) was significant (Appendix 1, Supplementary Digital Content, http://links.lww.com/MD/G988)).

The Hosmer–Lemeshow Chi-square statistic can be used to judge the goodness of fit of a model. A null hypothesis is adopted where the model is suitable when the *P* value of the Hosmer–Lemeshow Chi-square statistic is not significant. However, the *P* value of the Hosmer–Lemeshow Chi-square statistic in this study was significant. According to a previous study, if a study has more than 1000 subjects, the model is suitable even if the *P* value is significant.^20^ Thus, as this study had more than 1,000 subjects, the model could be suitable even if the *P* value is significant.

**Figure 1.**
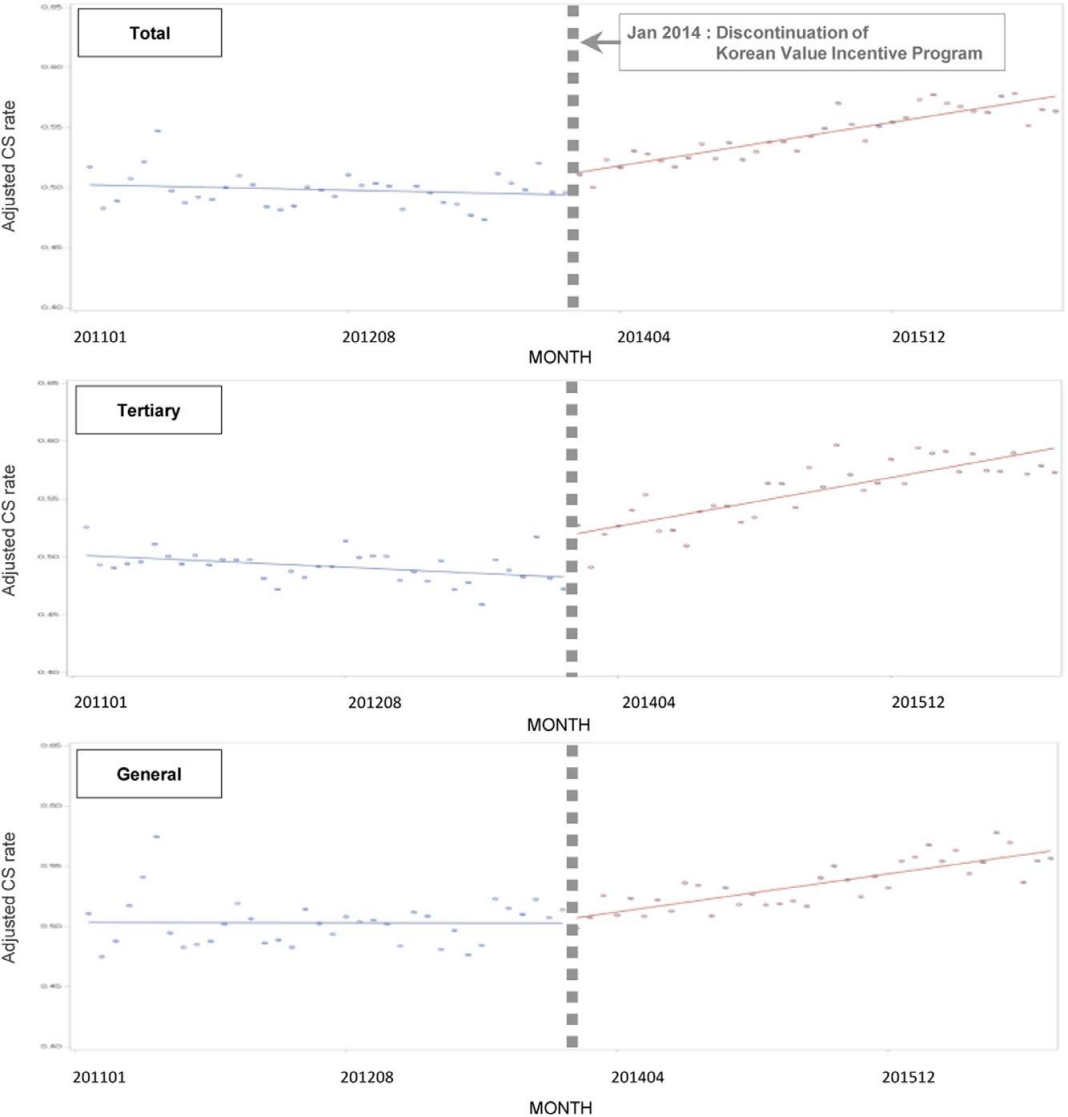
Interrupted time series analysis of risk-adjusted C-section rates before and after the end of the Korean Value Incentive Program.

The monthly average risk-adjusted C-section rates from 2011 to 2016 are shown in Figure 1. The difference in the monthly average risk-adjusted C-section rates before and after the end of the incentive program was evaluated based on hospital characteristics (Appendix 2, Supplemental Digital Content, http://links.lww.com/MD/G988).

C-section rates increased after the intervention (i.e., the end of the value incentive program) in hospitals of all types. The rate of change was 14.49% and 14.42% for tertiary and general hospitals, respectively (Table 1). For differences by administrative districts, changes of 8.12% in provinces and 11.89% in metropolitan were observed. According to the ownership type, changes of 11% in private and 3.80% in public hospitals were observed.

**Table 1:** Risk-adjusted C-section rates before and after the intervention (the end of the Korean Value Incentive Program).

Variables	N(%)	Preintervention period		Postintervention period		Difference	Changes (%)
		(Jan 2011–Dec 2013)		(Jan 2014–Dec 2016)			
		Mean	SD	Mean	SD		
Total	95(100.00)	46.36	9.00	51.38	10.37	5.02	10.83
Type							
Tertiary	41 (43.16)	47.81	9.25	54.74	11.09	6.93	14.49
General	54 (56.84)	45.16	8.99	51.67	11.06	6.51	14.42
District							
Province	43 (45.26)	48.95	8.89	52.93	11.84	3.98	8.12
Metropolitan	52 (54.74)	45.09	8.73	50.45	9.38	5.36	11.89
Ownership							
Private	89 (93.68)	46.13	8.95	51.21	10.53	5.08	11.00
Public	6 (6.32)	48.19	12.09	50.03	8.16	1.83	3.80

### 3.2. Interrupted time series analysis

Table 2 and Figure 1 show the interrupted time series analysis. The immediate effect of intervention (*β*_2_), a change of 1.73% (*P* < .05), was statistically significant, as was the trend after intervention (*β*_3_), at 0.21% (*P* < .001),. The slope ($\beta_{3}\;\;\;\;\;\;\;\;\;\;\;\;\;\;\beta_{1}$) showed an increase after the intervention to 0.25% per medical institution, which was contrary to the trend of the preintervention decline (negative slope).

**Table 2:** Results of interrupted time series analysis of risk-adjusted C-section rates before and after the end of Korean Value Incentive Program.

Variables	Coeff.	SE	95% CI		*P* value
			Lower	Upper	
Intercept ($\beta_{0}$)	51.68	1.46	48.81	54.55	<.0001
Baseline trend ($\beta_{1}$)	−0.04	0.05	−0.13	0.06	.08
Level change after policy ($\beta_{2}$)	1.73	0.85	0.06	3.39	.01
Trend change after policy ($\beta_{3}$)	0.21	0.07	0.09	0.34	<.0001
Type	−0.07	1.55	-3.81	2.26	.15
Tertiary					
(reference: General)					
District	−1.06	1.65	−4.29	2.18	.13
Metropolitan					
(reference: Province)					
Ownership	−0.49	1.32	−3.09	2.10	.26
Private (reference: Public)					

CI = confidence limit, SE = standard error.

The trend before intervention ($\beta_{1}$) and the hospital characteristics were not significant, as shown in Table 3. We expected that the effect of interventions would be clear if the analysis were performed based on hospital type, and further analysis was performed separately for tertiary hospitals and general hospitals (Table 3, Fig. 1).

**Table 3:** Results of interrupted time series analysis of risk-adjusted C-section rates before and after the end of Korean Value Incentive Program by hospital type.

Hospital type	Variable	Coeff.	SE	95% CI		*P* value
				Lower	Upper	
Tertiary	Intercept ($\beta_{0}$)	50.63	1.34	48.00	53.26	<.0001
	Baseline trend ($\beta_{1}$)	−0.08	0.04	−0.15	-0.00	<.05
	Level change	4.32	1.47	1.45	7.20	<.001
	after policy ($\beta_{2}$)					
	Trend change	0.27	0.07	0.12	0.41	<.001
	after policy ($\beta_{3}$)					
General	Intercept ($\beta_{0}$)	50.61	2.26	46.18	55.05	<.0001
	Baseline trend ($\beta_{1}$)	-0.02	0.09	−0.15	0.18	.64
	Level change	−0.36	0.88	−2.07	1.36	.38
	after policy ($\beta_{2}$)					
	Trend change	0.18	0.11	−0.03	0.39	<.05
	after policy ($\beta_{3}$)					

CI = confidence limit, SE = standard error.

In tertiary hospitals, the trends before intervention ($\beta_{1}$), the immediate effect of intervention ($\beta_{2}$), and the post-intervention trend ($\beta_{3}$) were all significant (*P* < .05). Before the intervention, a tendency toward a decrease, that is, a drop of 0.08%, was noted, and the immediate effect after the intervention was a relative increase of 4.32%. After the intervention, the rate gradually increased, by 0.27%. In tertiary hospitals, the risk-adjusted C-section rate tended to decrease before the intervention and then increased steadily immediately after the intervention.

However, in general hospitals, the regression coefficients before the intervention and the regression coefficient for the immediate effect of the intervention were not significantly different. Since the end of the Korean Value Incentive Program, the trend has increased significantly by 0.18%.

## 4. Discussion

The study analyzed the effects of the end of the Korean Value Incentive Program and the accompanying disclosure of the assessment results using the monthly average risk-adjusted C-section rates by hospitals. The study was conducted on tertiary and general hospitals with more than 200 births per year that were in operation from 2011 to 2016. Risk-adjusted C-section rates were calculated using the 364,352 claims generated by those institutions. The effect of the policy intervention was evaluated using an interrupted time series analysis.

The interrupted time series analysis has been proposed as a suitable method for identifying the effects of policy changes.^21^ To control the characteristics that do not change with the flow of time, it is appropriate to incorporate fixed effects.^22^ This study accounted for these using GEE. As all general and tertiary hospitals in Korea participated in the Korean Value Incentive Program,^4^ a case-control study design is not applied in this study.

Policy intervention would not be the only factor affecting the dependent variables of concern because healthcare policies have a wide variety of factors that affect healthcare provision. However, the ITS analysis can identify systematic changes in the dependent variables observed before and after the time interval in question, so it was adopted as a suitable method of analysis to assess the intervention effect.^19,23^

### 4.1. Statement of principal findings

Based on our results, after the Korean Value Incentive Program and disclosure of assessment results ended, the risk-adjusted C-section rates immediately increased and continued to increase thereafter.

According to the results of this interrupted time series analysis at tertiary and general hospitals, policy changes had different effects depending on hospital type. Tertiary hospitals responded to the policy change more sensitively than did general hospitals. The result could be explained by the defensive behavior of physicians in tertiary hospitals. A larger number of high-risk mothers visit tertiary hospitals for birth than visit general hospitals, and medical malpractice is more likely to occur with a higher number of deliveries.^24^ Previous studies showed that vaginal deliveries more commonly lead to guilty verdicts in medical disputes compared to cesarean deliveries.^25^

Our results support the conclusion that the selective behavior of providers on delivery was affected by the Korean Value Incentive Program (which was part of the performance compensation payment system) and the accompanying information disclosure. These findings are consistent with previous studies showing that C-section rates changed due to the publication of assessment results and previous studies showing that policy interventions affect the behavior of medical institutions.^5,7–9^

The C-section rates could be affected by the mother’s preferences for the mode of delivery.^26^ They may fear the way of normal delivery or the physical damage caused by vaginal delivery. Lee et al (2004) questioned 505 Korean women and found that women’s attitudes were not related to cesarean section surgery.^27^ They proposed that the preferences of healthcare practitioners and the healthcare system were the main factors in determining the mode of delivery. The study period (from 2011 to 2016) was not long enough to cause changes in the patients’ perspectives on the mode of delivery. The main cause of C-section rate changes will have come from the changes in hospital behavior.

### 4.2. Implications for policy, practice, and research

According to OECD health statistics, the C-section rate in Korea in 2017 was 45.2%, far exceeding the average rate of other OECD countries. Thus, policy intervention is required. Since interventions addressing C-section rates have been discontinued at this time, management incentives are required for quality assessment.

First, to resume quality assessment, a risk-adjustment model must be developed that can be applied to the DRG payment system, which is one of the reasons that quality assessment for cesarean delivery was terminated. This model must determine whether the assigned KDRG fully reflects the patient’s clinical status.

Second, an improved evaluation method is required. In Korea, pay-for-performance (P4P) programs adopt a relative evaluation method based on a ranking of hospitals. A large number of medical institutions oppose the system because of the uncertainty it raises about the potential increases or decreases in payments based on these evaluations.^28^ Transition from a performance payment system to an absolute evaluation method will likely be accepted as a means of acknowledging the doctors’ expertise rather than as a mechanism of control over the provider.^29^ Previous studies have shown that the absolute evaluation method was more effective than the relative evaluation method.^30,31^

Third, while publicizing assessment results is important, the public will need to easily access and understand the information. In addition, an effective disclosure strategy for the assessment results must be used when selecting medical providers.^32,33^ A previous study showed that when the rates were publicized through mass media outlets, there was a reduction in C-section rates relative to other periods.^6^

This study is meaningful in that it is an empirical study that evaluated the effect of the end of discontinuation of the Korean Value Incentive Program on the quality level of medical services by calculating the index which is evaluated using the risk-adjusted cesarean delivery rate as a dependent variable. In addition, to compensate for the short analysis period, which was suggested as a limitation in previous studies, and the impact could not be confirmed due to the lack of a control group, a total of 6 years were analyzed. However, since this study is not an analysis including a control group, there is a limit to estimating the pure effect of the policy only for the Korean Value Incentive Program. In particular, it is difficult to affirm the effect of the performance of the incentive system or information disclosure only, because the incentive system was carried out in conjunction with the disclosure of the evaluation results.

Another limitation of the study is that the uncertainty that does occur by using the risk-adjusted C-section rates was not considered. This study was performed according to previous studies^16^ that the coefficients calculated by pooling individual estimates using inverse variance weights and the coefficients calculated through average estimates for each hospital unit yielded consistent results. Therefore, it would be better to consider these in further studies.

## 5. Conclusion

This study showed that C-section rates have steadily increased since the discontinuation of the Korean Value Incentive Program and the associated disclosure of assessment results. This represents a return to the selective behavior of physicians on delivery from before the implementation of the program due to the absence of financial and social incentives.

To manage the increasing C-section rates, new policy intervention may be implemented. However, rather than applying for the previous assessment program, it should be modified to reflect environmental changes, such as the adoption of KDRG and the use (and publicizing) of a new evaluation method. Successful implementation of the next-generation evaluation program will require discussion with diverse stakeholders in the medical community and will require a reasonable compensation system.

**Key points**

- This study investigated how hospitals responded to terminating the Korean Value Incentive Program for cesarean delivery.
- Risk-adjusted C-section rates increased immediately after the discontinuation? of value incentive program.
- Tertiary hospitals showed greater increases in C-section rates than general hospitals after the intervention.

## Acknowledgments

The authors would like to sincerely thank the institute of health and welfare, Yonsei University who allowed us to partake in this study to share their expert knowledge on the Korean Value incentive program. The English in this document has been checked by at least two professional editors, both native speakers of English. For a certificate, please see http://www.textcheck.com/certificate/Xvf6L2. This study was prepared by partially revising and supplementing the first author's master's thesis.

## Author contributions

PYH and LKS designed the study. KJH performed the literature review and interpretation for data analysis. PYH and LKS analyzed the data. PYH and LKS wrote the draft. ALL authors read and approved the final manuscript.

## Supplementary Material


