# Supplementary material for: Changes in cesarean section rate before and after the end of the Korean Value Incentive Program
Source: Medicine (Baltimore). 2022 Aug 19;101(33):e29952. doi: 10.1097/MD.0000000000029952 (PMC9388010; doi:10.1097/MD.0000000000029952)
Supplement: Supplementary file 1 [file medi-101-e29952-s001.pdf]

**Supplemental Digital Content(Appendix 1) for**

# Changes on Cesarean Section Rate before and after the End of Korean Value Incentive Program

YouHyun Park, MPH[1], Jae-hyun Kim, PhD[2], Kwang-soo Lee, PhD [1,§]

[1]Department of Health Administration, Graduate School, Yonsei University, Seoul, Republic of Korea

[2]Department of Healthcare Administration, Dankook University, Cheonan, Republic of Korea

§ **Corresponding Author** Kwang-soo Lee, PhD, MPH

Department of Health Administration, Yonsei University, Wonju, Gwangwondo, Republic of Korea

Tel : +82-33-760-2426 Email : planters@yonsei.ac.kr

**Supplemental Digital Content (Appendix 1). Risk factors included in the risk-adjustment model**

| Risk Factors | | Vaginal Delivery | | Cesarean Section | | p-value | Odds Ratio |
|---|---|---|---|---|---|---|---|
| | | N | % | N | % | | |
| Breech malpresentation | Yes | 2,182 | 5.81 | 35,378 | 94.19 | <0.001 | 56.03 |
| | No | 185,912 | 56.89 | 140,880 | 43.11 | | |
| Pre-eclampsia | Yes | 6,323 | 29.07 | 15,426 | 70.93 | <0.001 | 4.99 |
| | No | 181,771 | 53.06 | 160,832 | 46.94 | | |
| Malignancy | Yes | 268 | 41.61 | 376 | 58.39 | <0.001 | 2.76 |
| | No | 187,826 | 51.64 | 175,882 | 48.36 | | |
| Placenta previa | Yes | 1,125 | 6.21 | 16,986 | 93.79 | <0.001 | 55.15 |
| | No | 186,969 | 54.00 | 159,272 | 46.00 | | |
| Multiple pregnancy | Yes | 3,140 | 62.93 | 1,850 | 37.07 | <0.001 | NA |
| | No | 184,954 | 51.47 | 174,408 | 48.53 | | |
| Cephalopelvic disproportion | Yes | 17,342 | 13.80 | 108,293 | 86.20 | <0.001 | 15.31 |
| | No | 170,752 | 71.53 | 67,965 | 28.47 | | |
| Fetal stress | Yes | 22,815 | 61.67 | 14,180 | 38.33 | <0.001 | NA |
| | No | 165,279 | 50.49 | 162,078 | 49.51 | | |
| Maternal age | Yes | 54,494 | 42.82 | 72,772 | 57.18 | <0.001 | 1.31 |
| | No | 133,600 | 56.35 | 103,486 | 43.65 | | |
| Bleeding | Yes | 304 | 29.26 | 735 | 70.74 | <0.001 | 2.20 |
| | No | 187,790 | 51.69 | 175,523 | 48.31 | | |
| Cord prolapse | Yes | 7,537 | 70.32 | 3,181 | 29.68 | <0.001 | NA |
| | No | 180,557 | 51.06 | 173,077 | 48.94 | | |
| Diabetes | Yes | 11,205 | 45.09 | 13,645 | 54.91 | <0.001 | 1.10 |
| | No | 176,889 | 52.10 | 162,613 | 47.90 | | |
| Fetal abnormalities | Yes | 19,290 | 42.60 | 25,995 | 57.40 | <0.001 | 1.83 |
| | No | 168,804 | 52.91 | 150,263 | 47.09 | | |
| Oligohydramnios /Polyhydramnios | Yes | 8,508 | 45.41 | 10,229 | 54.59 | <0.001 | 1.47 |
| | No | 179,586 | 51.96 | 166,029 | 48.04 | | |
| Premature rupture of membranes | Yes | 51,748 | 68.08 | 24,265 | 31.92 | <0.001 | NA |
| | No | 136,346 | 47.29 | 151,993 | 52.71 | | |
| Previous cesarean section | Yes | 2,064 | 3.29 | 60,635 | 96.71 | <0.001 | 24.48 |
| | No | 186,030 | 61.67 | 115,623 | 38.33 | | |
| Preterm delivery | Yes | 19,311 | 39.91 | 29,072 | 60.09 | <0.001 | 1.71 |
| | No | 168,783 | 53.42 | 147,186 | 46.58 | | |
| Sexually transmitted disease | Yes | 45 | 32.37 | 94 | 67.63 | <0.001 | 9.12 |
| | No | 188,049 | 51.63 | 176,164 | 48.37 | | |
| $R^2$ | | | | | | | 0.67 |
| C-statistic | | | | | | | 0.91 |
| Hosmer–Lemeshow test, $X^2$(p-value) | | | | | | | 4,472.80 (<.0001) |

†CI: confidence limit, NA: not available

Supplemental Digital Content (Appendix 2). Monthly average of risk-adjusted C-section rates in hospitals (%)

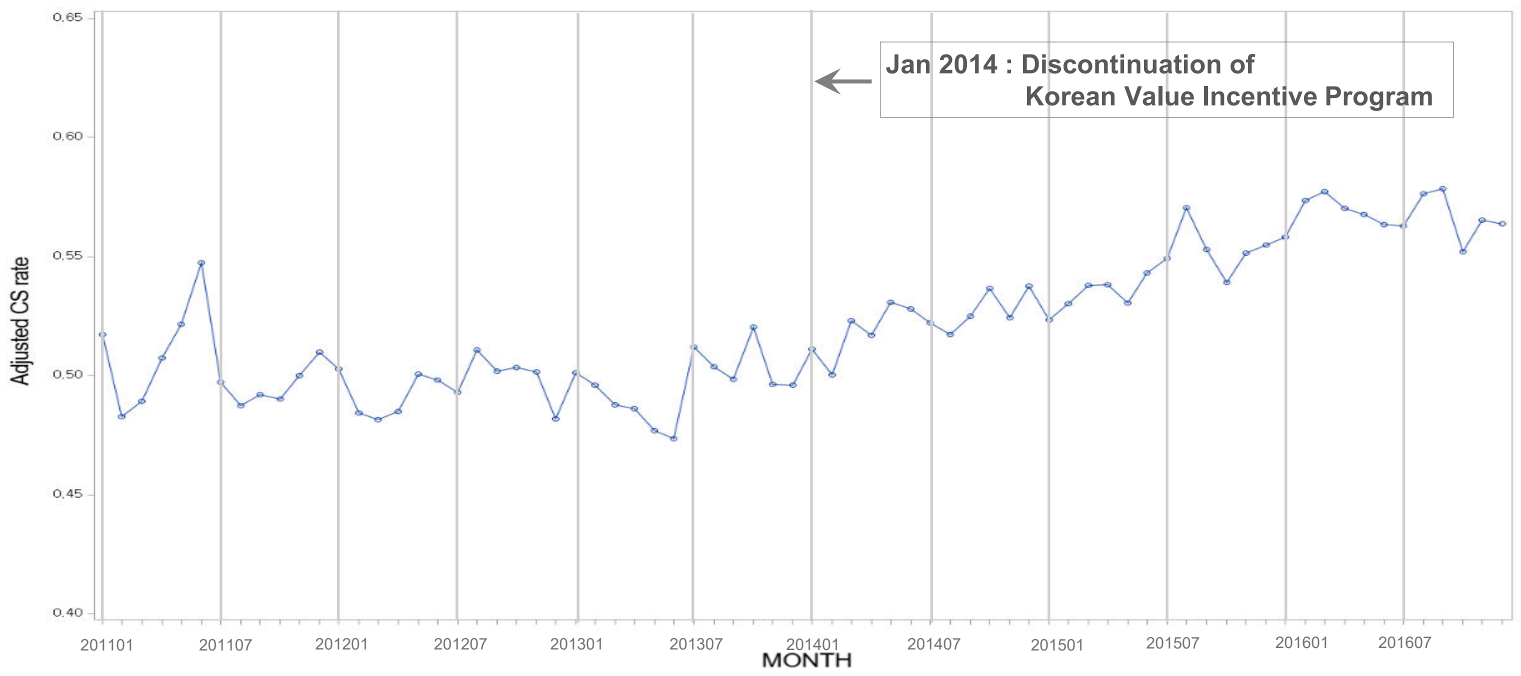

**Supplemental Digital Content (Appendix 3) for**

# Changes on Cesarean Section Rate before and after the End of Korean Value Incentive Program

YouHyun Park, MPH[1], Jae-hyun Kim, PhD[2], Kwang-soo Lee, PhD [1,§]

[1]Department of Health Administration, Graduate School, Yonsei University, Seoul, Republic of Korea

[2]Department of Healthcare Administration, Dankook University, Cheonan, Republic of Korea

§ **Corresponding Author** Kwang-soo Lee, PhD, MPH

Department of Health Administration, Yonsei University, Wonju, Gwangwondo, Republic of Korea

Tel : +82-33-760-2426 Email : planters@yonsei.ac.kr

**Supplemental Digital Content (Appendix 3): The autocorrelation between observations**

The assumption of the standard regression model is that the observations are independent. In time series analysis, these assumption is often violated because observations that are measured at time points that are close tend to be similar than that are further. Failure to correct this autocorrelation can lead to underestimation of the standard error and overestimation of the impact of the intervention[1]. In epidemiology, autocorrelation is explained by other variables such as seasonality, but after controlling these factors, residual autocorrelation is known to be rarely a problem [2].

There was no seasonality in the C-section rates (Supplemental Digital Content (Appendix 2)), but there was an autocorrelation. Therefore, GEE model (the use of proc genmod in SAS), was chosen for the analysis to allow for heterogeneity in residual variance among the two phases, and estimation of the Autoregressive of first order(AR1) autocorrelation parameter. In other words, the structure of the covariance matrix was specified as AR1, which assume that the interval length is the same between any two observations [3].

Reference


1. Wagner AK, Soumerai SB, Zhang F, Ross-Degnan D. Segmented regression analysis of interrupted time series studies in medication use research. *Journal of clinical pharmacy and therapeutics.* 2002;27(4):299-309.
2. Bernal JL, Cummins S, Gasparrini A. Interrupted time series regression for the evaluation of public health interventions: a tutorial. *International journal of epidemiology.* 2017;46(1):348-355.
3. Smith T, Smith B. PROC GENMOD with GEE to analyze correlated outcomes data using SAS. *San Diego (CA): Department of Defense Center for Deployment Health Research, Naval Health Research Center.* 2006.
